# ANP and BNP Exert Anti-Inflammatory Action via NPR-1/cGMP Axis by Interfering with Canonical, Non-Canonical, and Alternative Routes of Inflammasome Activation in Human THP1 Cells

**DOI:** 10.3390/ijms22010024

**Published:** 2020-12-22

**Authors:** Letizia Mezzasoma, Vincenzo Nicola Talesa, Rita Romani, Ilaria Bellezza

**Affiliations:** Department of Medicine and Surgery, University of Perugia, 06123 Perugia, Italy; vincenzo.talesa@unipg.it (V.N.T.); rita.romani@unipg.it (R.R.); ilaria.bellezza@unipg.it (I.B.)

**Keywords:** Natriuretic Peptide Receptor, cGMP-dependent protein kinase, IL-1β, inflammation, immune-modulation

## Abstract

Dysregulated inflammasome activation and interleukin (IL)-1β production are associated with several inflammatory disorders. Three different routes can lead to inflammasome activation: a canonical two-step, a non-canonical Caspase-4/5- and Gasdermin D-dependent, and an alternative Caspase-8-mediated pathway. Natriuretic Peptides (NPs), Atrial Natriuretic Peptide (ANP) and B-type Natriuretic Peptide (BNP), binding to Natriuretic Peptide Receptor-1 (NPR-1), signal by increasing cGMP (cyclic guanosine monophosphate) levels that, in turn, stimulate cGMP-dependent protein kinase-I (PKG-I). We previously demonstrated that, by counteracting inflammasome activation, NPs inhibit IL-1β secretion. Here we aimed to decipher the molecular mechanism underlying NPs effects on THP-1 cells stimulated with lipopolysaccharide (LPS) + ATP. Involvement of cGMP and PKG-I were assessed pre-treating THP-1 cells with the membrane-permeable analogue, 8-Br-cGMP, and the specific inhibitor KT-5823, respectively. We found that NPs, by activating NPR-1/cGMP/PKG-I axis, lead to phosphorylation of NLRP3 at Ser295 and to inflammasome platform disassembly. Moreover, by increasing intracellular cGMP levels and activating phosphodiesterases, NPs interfere with both Gasdermin D and Caspase-8 cleavage, indicating that they disturb non-canonical and alternative routes of inflammasome activation. These results showed that ANP and BNP anti-inflammatory and immunomodulatory actions may involve the inhibition of all the known routes of inflammasome activation. Thus, NPs might be proposed for the treatment of the plethora of diseases caused by a dysregulated inflammasome activation.

## 1. Introduction

Natriuretic Peptides (NPs) are a family of structurally related cardiac hormones that include Atrial Natriuretic Peptide (ANP) and B-type Natriuretic Peptide (BNP). ANP and BNP share a common receptor, the Natriuretic Peptide Receptor-1 (NPR-1) [1]. It is composed of an extracellular ligand binding domain, a single membrane-spanning region and an intracellular domain comprising a carboxyl-terminal guanylyl cyclase domain. Upon binding with ANP or BNP, NPR-1 signals by catalyzing the synthesis of cyclic guanosine monophosphate (cGMP). The increase in intracellular cGMP levels can stimulate cGMP-dependent phosphodiesterases, cGMP-dependent protein kinases (PKG-I and -II), and cGMP-dependent ion channels [2].

ANP is predominantly secreted from the heart in response to atrial stretch and its expression increases in response to elevated atrial pressure [3]. BNP, secreted from both atria and ventricles, is increased in patients with congestive heart failure and acts as a defense against ventricular overload [4].

The protective action of ANP and BNP depends on their ability to promote diuresis and natriuresis, to induce vasodilation and to regulate systemic blood pressure and body fluid balance [2,5]. Furthermore, ANP and BNP act on cardiac cells themselves to counteract cardiac hypertrophy and fibrosis [6]. Synthetic ANP (Anaritide or Carperitide) and BNP (Nesiritide) have been studied for their therapeutic role in hypertension and heart failure [3]. Besides the known cardiac functions, NPs has been shown to control fatty acid mobilization and cellular energy metabolism in adipocytes and skeletal myocytes [3], pointing to pleiotropic effects not confined to cardiac and renal tissues. 

NPs and their receptors are expressed in many cells and tissues, including immune ones, where they exert multiple effect in both physiological and pathological conditions. In fact, a role for natriuretic peptides in inflammation has been depicted. It has been thoroughly demonstrated that inflammation increases the expression natriuretic peptides [7] and that NPR-1 is involved in immune and inflammatory reactions [8]. In particular, ANP and BNP secretion is modulated by angiotensin II, thyroid hormones, interleukin (IL)-1, IL-6, and tumor necrosis factor (TNF)-α [5,9,10,11]. However, both pro-inflammatory and anti-inflammatory effects have been reported so far. NPs are frequently elevated among critically ill patients and might play a role in cardiopulmonary dysfunction, sepsis and septic shock [9,12]. Moreover, the genetic ablation of NRP-1 has been associated with a reduction in antigen-induced pulmonary inflammation [13] and allergic inflammation [14] suggesting a pro-inflammatory role for the NPs. On the contrary, NRP-1 knock out mice show an increase in renal [15] and cardiac [16,17] Nuclear factor kappa B (NF-κB) activation, indicating anti-inflammatory properties for NPs. Therefore, the role of NPs in inflammation is still incompletely understood.

In the complex scenario of inflammation, a pivotal role is played by the inflammasome, a cytoplasmic supramolecular platform devoted to detecting pathogen associated molecular patterns (PAMPs) and damage associated molecular patterns (DAMPs) and deputed to the activation of the highly pro-inflammatory cytokines IL-1β and IL-18. The inflammasome includes several molecular components: a sensor receptor, an adaptor protein (Apoptosis-associated speck-like protein containing a CARD, ASC), and an effector enzyme (Caspase-1). The cytoplasmic sensor receptor family include the nucleotide-binding and oligomerization domain (NOD)-like receptors (NLRs) family, composed of at least 22 members [18,19], the most studied of which is the NLRP3. When NLRs detect PAMPs or DAMPs, they enroll the adaptor protein ASC, rendering it capable of recruiting pro-Caspase-1 which completes the formation and activation of the canonical inflammasome [19,20]. 

Maturation and secretion of IL-1β by NLRP3 inflammasome requires a two-step mechanism. The priming step entails the stimulation of toll-like receptors (TLRs) by appropriated stimuli, such as lipopolysaccharide (LPS) from gram negative bacteria. This event leads to an NF-κB-dependent upregulation of cellular NLRP3 and *pro-IL-1β* transcription. In the activation step PAMPs and DAMPs, binding to NLRP3, trigger the assembly of the inflammasome platform though combined events such as ion flux, mitochondrial and lysosomal dysfunction, and reactive oxygen species (ROS) generation [19].

Given the potent inflammatory action of IL-1β, inflammasome activation is tightly controlled to prevent or limit collateral damage to the host [19]. NLRP3 inflammasome is regulated in both the priming and activation steps. The regulation of the priming step typically entails the activation of the transcription factor NF-κB. The regulation of the activation step affects the oligomerization of the inflammasome components via several post-translational modifications including phosphorylation and ubiquitination. Different phosphorylation sites in NLRP3 have been proposed to modulate inflammasome activation either positively (Ser194 and Ser295) or negatively (Ser5, Ser295, and Y861) [19]. Besides this canonical pathway, a non-canonical and an alternative route of inflammasome activation have been described. The non-canonical inflammasome activation is triggered by Caspase-4 and -5 and does not require a priming step, while the alternative inflammasome activation involves Caspase-8 cleavage [20]. Regardless the activation route, all the inflammasome activation pathways culminate in IL-1β maturation and secretion.

The dysregulation of inflammasome activation and IL-1β production has been associated to a plethora of inflammatory disorders, including neurodegenerative diseases, cancer, autoinflammatory disorders, and cardiometabolic diseases [21].

Our research group unraveled the link between inflammasome activation and natriuretic peptides [22,23]. We have shown that both ANP and BNP act as potent anti-inflammatory and immunomodulatory factors by inhibiting NLRP3 activation and IL-1β secretion in human monocytic cells. In particular, the natriuretic peptides, acting via NF-κB and extracellular signal-regulated kinases (ERK1/2), reduce the mature/active form of Caspase-1 thus reducing maturation and secretion of IL-1β.

In the present study, we identified the molecular mechanism associated to NPs-mediated inhibition of inflammasome activation and described, for the first time, NPR1/cGMP/PKG-I axis involvement in the control of inflammasome activation. We showed that, by activating PKG-I, ANP and BNP lead to the phosphorylation and disassembly of the inflammasome platform. Moreover, we also showed that ANP and BNP interfere with the alternative and the non-canonical routes of inflammasome activation. These findings support a potential therapeutic use of natriuretic peptides for inflammasome-driven inflammatory and autoimmune diseases.

## 2. Results and Discussion

### 2.1. cGMP/PKG-I Axis Drives ANP/BNP Inhibitory Effect on IL-1β Secretion

**Figure 1:**
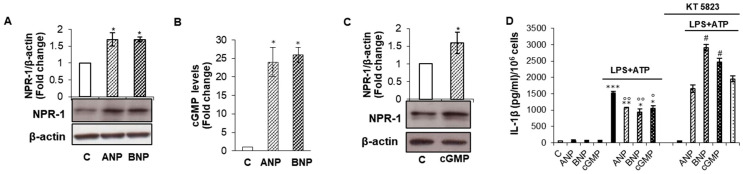
Cyclic Guanossine monophosphate (cGMP)/Protein kinase G—I (PKG-I) axis drives Atrial natriuretic peptide (ANP)/ B-type natriuretic peptide (BNP) inhibitory effect on Interleukin (IL)-1β secretion. THP-1 cells were treated with ANP or BNP (10^−8^ M) (**A**,**B**) or 8-Br-cGMP (100 µM) (**C**) for 1 h (**A**,**C**) or 30 min (**B**). (**A**,**C**) NPR-1 protein expression by Western blot. Protein loading was assessed by re-probing the blots with an anti-β-actin antibody. Western blots are representative of *n* = 3 independent experiments. (**B**) Cells were lysed, and cGMP intracellular levels were measured by ELISA assay (basal cGMP: 64 fmol/10^6^ cells). (**D**) THP-1 cells, either pretreated for 10 min with ANP or BNP (10^−8^ M) or 8-Br-cGMP (100 µM) or left untreated, were exposed to LPS (10 µg/mL) for 20 min and treated with ATP (5 mM) for 48 h. When used, KT-5823 (1μM) was added 1 h before ANP, BNP, or cGMP. The supernatants were collected and IL-1β release was measured by ELISA. All histograms indicate mean ± SD of at least *n* = three independent experiments, each one tested in triplicate. * *p* < 0.05, ** *p* < 0.01, *** *p* < 0.001 versus untreated control cells; ° *p* < 0.05, °° *p* < 0.01, versus LPS + ATP-treated cells; # *p* < 0.05 versus KT-5823 + LPS + ATP treated cells.

Both natriuretic peptides (NPs) ANP and BNP promote their biological functions through natriuretic peptide receptor 1 (NPR-1). Upon binding and dimerization, a conformational change activates the guanylyl cyclase domain of the receptor, resulting in an elevation of intracellular cGMP levels [2]. We first analyzed the effects of ANP and BNP on NPR-1 and cGMP levels in THP-1 cells, by evaluating NPR-1 protein expression and cGMP intracellular levels after a 30 min treatment with ANP or BNP (10^−8^ M). We found that both ANP and BNP induce a significant increase in NPR-1 protein expression (Figure 1A) and in cGMP intracellular levels (Figure 1B), indicating NPs capability to increase NPR1 expression and to activate NPR-1/cGMP axis in THP-1 cells.

Cyclic-GMP cascade is believed to be the key event eliciting ANP and BNP mediated cellular and physiological responses [2]. Therefore, we asked whether cGMP might drive NP-mediated effects in THP-1 cells. To address this hypothesis, we evaluate whether 8-Br-cGMP, a cell membrane permeable cGMP analogue, could increase NPR-1 expression. We found that the exposure of THP-1 cells to 8-Br-cGMP (100 μM) for 1 h increases NRP-1 expression of the same order of magnitude as NPs (Figure 1C), confirming that the intracellular rise in cGMP levels is at the basis of NPs-mediated effects in THP-1 cells.

We previously demonstrated that both NPs elicit an inhibitory action on LPS plus ATP-induced IL-1β secretion in THP-1 cells and that a 48 h treatment was the most suitable time point to analyze IL-1β production [22,23]. To verify whether the anti-inflammatory effects of NPs could rely on intracellular cGMP, THP-1 cells were pre-treated for 10 min with ANP, BNP and 8-Br-cGMP, before LPS (10 μg/mL) and ATP (5 mM) (LPS + ATP) stimulation for 48 h. We found that 8-Br-cGMP treatment inhibits LPS + ATP-induced IL-1β secretion, mirroring the effects of both ANP and BNP treatment (Figure 1D). These data demonstrate a direct inhibitory action of 8-Br-cGMP on LPS + ATP-induced IL-1β secretion, and strongly suggest that cGMP mediates ANP and BNP inhibitory effects on IL-1β secretion. Because cGMP-dependent protein kinase I (PKG-I) is an important downstream effector of cGMP [2], we hypothesized that this protein kinase could be involved in the observed responses. To address this hypothesis, THP-1 cells were pre-treated for 1 h with KT-5823 (1 μM), a selective PKG-I inhibitor, before LPS + ATP stimulation for 48 h, in the presence or absence of ANP, BNP, and 8-Br-cGMP. We found that KT-5823 completely abrogates NPs and 8-Br-cGMP inhibitory effect on IL-1β secretion (Figure 1D); thus, suggesting a direct involvement of PKG-I on both ANP- and BNP-mediated effects. Moreover, KT-5823 did not affect IL-1β production neither in basal conditions nor upon LPS + ATP stimulation. All together, these data strongly indicate that ANP and BNP, via NPR-1, exert anti-inflammatory action through the cGMP/PKG-I signaling pathway. 

### 2.2. cGMP/PKG-I Axis is Involved in the Priming Mechanism Controlling IL-1β Production 

**Figure 2:**
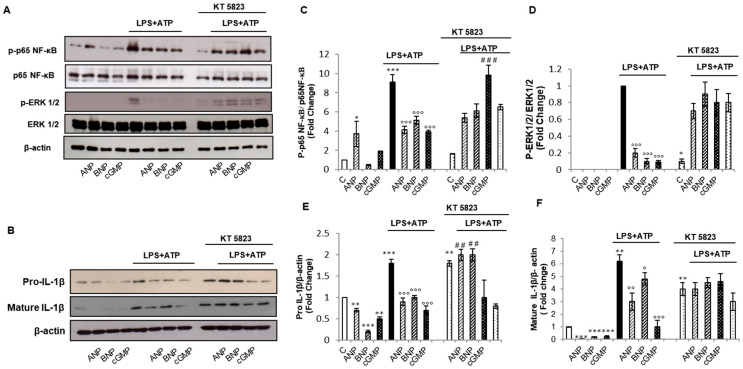
cGMP/PKG-I axis Is Involved in the priming mechanism controlling IL-1β production**.** THP-1 cells were pre-treated for 10 min with ANP (10^−8^ M) or BNP (10^−8^ M) or cGMP (100 µM 8-Br-cGMP) in the absence or presence of LPS (10 µg/mL for 20 min) + ATP (5 mM for 40 min). When KT-5823 (1 μM) was used, it was added 1 h before ANP, BNP, or 8-Br-cGMP treatment. Cell lysates were immunoblotted for p-p65-NF-κB and total p65-NF-κB, p-ERK 1/2 and total ERK1/2 (**A**) or pro- and mature-IL-1β (**B**). Protein loading was assessed by re-probing the blots with an anti-β-actin antibody. Representative western blots images are shown. (**C**–**F**) Histograms of densitometric quantification. Bars represent the ratio between phosphorylated and total protein band intensity. Untreated cells were used as control and assumed as 1. When no band appears in control cells, LPS + ATP was assumed as 1. All histograms indicate the mean ± SD of at least *n* = three independent experiments, each one tested in triplicate. * *p* < 0.05, ** *p* < 0.01, *** *p* < 0.001 versus untreated cells; ° *p* < 0.05, °° *p* < 0.01, °°° *p* < 0.001 versus LPS + ATP treated cells; ## *p* < 0.01, ### *p* < 0.001 versus KT-5823 + LPS + ATP treated cells.

The potent pleiotropic actions of IL-1β rely upon a tight control of its synthesis and maturation by a two steps mechanism: a priming step and an activation step. The priming step consists in the transcription of the *IL-1β* gene upon engagement of the toll-like-receptors (TLRs) by specific ligands, including bacterial lipopolysaccharides (LPS). TLRs engagement culminates in the activation of the transcription factor NF-κB which positively controls the expression of *pro-IL-1β* gene. Moreover, mitogen-activated protein kinase (MAPK) cascade is another crucial pathway that, through TLR signaling, can regulate IL-1β production [24]. We have previously demonstrated that both ANP and BNP control IL-1β production, by inhibiting NF-κB and ERK 1/2 activation [22,23]. To further explore the mechanism of action of ANP and BNP, we evaluated the involvement of cGMP and its downstream effector PKG-I, on NPs-induced inhibition of NF-κB and ERK1/2. THP-1 cells were pre-treated for 10 min with ANP, BNP or 8-Br-cGMP, before LPS + ATP stimulation for 1 h. We found that 8-Br-cGMP treatment, inhibits LPS + ATP-induced NF-κB (Figure 2A,C) and ERK1/2 phosphorylation (Figure 2A,D). The inhibition of NF-κB and ERK1/2 phosphorylation culminates in the reduction of pro-IL-1β protein expression (Figure 2B,E). The action of 8-Br-cGMP is superimposable to that of ANP or BNP (Figure 2A–E). These results are in agreement with previously published reports showing that ANP and BNP strongly reduced the phosphorylation of NF-κB inhibitor Iκ-Bα [22,23].

A 1 h KT-5823 pre-treatment reverts ANP and BNP inhibitory effect on LPS + ATP-induced NF-κB (Figure 2A,C) and ERK1/2 phosphorylation (Figure 2A,D) and on pro-IL-1β expression (Figure 2B,E). KT-5823 also counteracts 8-Br-cGMP effects on NF-κB (Figure 2A,C) and ERK1/2 phosphorylation (Figure 2A,D). We should note that the exposure to KT-5823 slightly increases NF-κB phosphorylation and the related pro-IL-1β expression (Figure 2A–C,E). These results correlate with data in the literature reporting that cGMP/PKG axis mediated the downregulation of NF-κB p65 phosphorylation leading to the inhibition of CC chemokime ligand 5 (CCL5) secretion by CD8+ T cells [25]. Furthermore, it has also recently been reported that the xanthine and piperazine derivative, 7-[2-[4-(2-Chlorophenyl)piperazinyl]ethyl]-1,3-dimethylxanthine (KMUP-1), inhibits ischemia-induced apoptosis in H9c2 embryonic rat heart-derived cell line via upregulation of PKG and reduced activation of ERK1/2 [26]. We should note that the expression levels of ERK1/2 and NF-κB p65 remain unchanged in all the tested conditions strongly suggesting that phosphorylation events are at the basis of ANP and BNP-induced effects. All together, these data strongly suggest that ANP and BNP modulate the priming mechanism responsible for IL-1β production by acting through the cGMP/PKG-I axis that leads to the inhibition of NF-κB and ERK1/2 phosphorylation. 

To exert its biological effects pro-IL-1β needs to be converted in the mature form through a proteolytical cleavage. Besides confirming that both ANP and BNP inhibit pro-IL-1β cleavage (Figure 2B,F) [22,23], we observed that 8-Br-cGMP mirrors NPs effects (Figure 2B,F). However, it is conceivable that, at least at the early time-points used in this experimental condition, BNP displays a lower efficacy in the conversion of pro-IL-1β into the mature form compared to ANP and 8-Br-c-GMP (Figure 2B,E,F). We should note that the significant reduction in IL-1β secretion (Figure 1D) might result from the inhibitory effects of ANP, BNP and 8-Br-cGMP on maturation of IL-1β (Figure 2B,F). In the presence of KT-5823 neither NPs nor 8-Br-cGMP elicit any downregulatory effect on pro-and mature IL-1β compared to KT-5823 + LPS + ATP (Figure 2B,E,F). In agreement, the presence of KT-5823 also abrogates the inhibitory effects of ANP, BNP, and 8-Br-cGMP on IL-1β secretion (Figure 1D). The discrepancies in the relative amounts of intracellular pro- and mature IL-1β compared to the secreted form may depend on time-point and method of assessment. In fact, the intracellular levels of pro-and mature IL-1β are analyzed by western blotting 1 h after LPS + ATP addition whereas, IL-1β secretion is analyzed in culture medium after a 48 h LPS + ATP exposure. Nevertheless, a contribution of pro-IL-1β expression levels on the amount of secreted IL-1β cannot be ruled out. These data suggest that NPs through the cGMP/PKG-I axis also act on the activation step needed for mature IL-1β production. 

### 2.3. cGMP/PKG-I Axis Is Involved in the Activation Step of IL-1β Maturation 

The activation step needed for pro-IL-1β processing requires the proteolytic activation of pro-Caspase-1 by the inflammasome platform. The inflammasome is an intracellular macromolecular signaling platform composed of cytoplasmatic pattern recognition receptors (PRRs), the adaptor protein ASC and pro-Caspase-1 [19,20]. PRRs are intracellular sensors deputed to the recognition and binding of cellular distress [18]. NLRP1, the first inflammasome receptor discovered, is activated by a pathogen-mediated functional degradation [27]. NLRP3 responds to a diverse set of stimuli including particulate matter, pore-forming toxins, RNA-DNA hybrids, and, as used in our experimental settings, extracellular ATP [28]. The absent in melanoma 2 (AIM2) PRR recognizes cytosolic DNA, including mitochondrial DNA [29].

**Figure 3:**
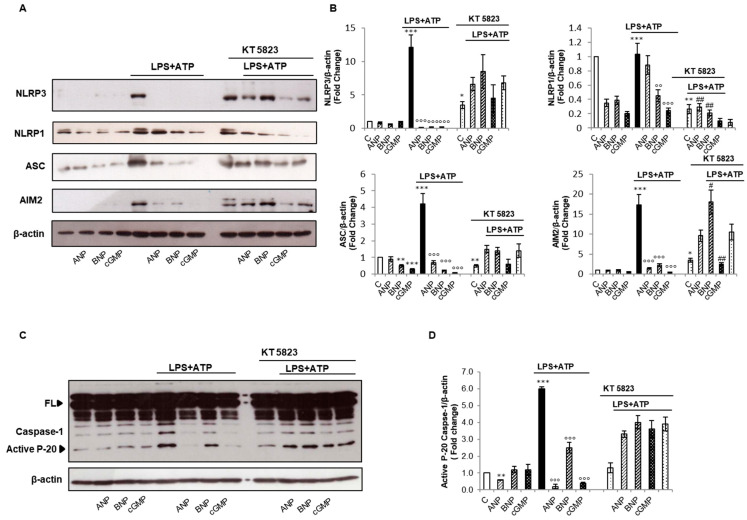
cGMP/PKG-I axis is involved in the activation step of IL-1β maturation. THP-1 cells were pre-treated for 10 min with ANP (10^−8^ M) or BNP (10^−8^ M) or cGMP (100 µM 8-Br-cGMP) in the absence or presence of LPS (10 µg/mL for 20 min) + ATP (5 mM for 40 min). When KT-5823 (1 μM) was used, it was added 1 h before ANP, BNP, or 8-Br-cGMP treatment. Cell lysates were immunoblotted for NLRP3, NLRP1, ASC, AIM2 (**A**) or Caspase-1 (**C**). The blots were stripped and re-probed with mouse anti-β-actin to confirm equal loading. Representative western blots images are shown. (**B**,**D**) Histograms of densitometric quantification. Bars represent the ratio between respective protein and β-actin band intensity. Untreated cells were used as control and assumed as 1. The histogram indicates mean ± SD of at least *n* = three independent experiments, each one tested in triplicate. * *p* < 0.05, ** *p* < 0.01, *** *p* < 0.001 versus untreated cells; °° *p* < 0.01, °°° *p* < 0.001 versus LPS + ATP treated cells; # *p* < 0.05 and ## *p* < 0.01 versus KT-5823 + LPS + ATP treated cells.

We have previously demonstrated that both ANP and BNP strongly interfere with NLRP3-ASC-Caspase-1 activation [22,23]. To further explore the molecular mechanism of ANP- and BNP- mediated effects, we analyzed the involvement of the cGMP/PKG-I axis on inflammasome activation. We found that ANP, BNP, and 8-Br-cGMP pre-treatment strongly inhibit LPS+ATP-induced expression of the elements of the inflammasome platform, i.e., NLRP3, NLRP1, AIM2, and ASC (Figure 3A,B). NF-κB signaling and MAPK cascade are both crucial pathways that, through TLR signaling, can regulate PRRs expression [24]. Our results are in agreement with the finding that BAY 11-7082, a specific NF-κB inhibitor, mimics ANP and BNP effects on NLRP3 [22,23]. Furthermore, the blockade of ERK1 significantly inhibits the rapid priming of NLRP3 in monocytes [30] and the inhibition of ERK1/2 with the specific MAPKK-1 inhibitor, U0126, mirrors BNP induced effects on NLRP3 expression [23].

Here we showed that KT-5823 pre-treatment completely reverts ANP and BNP inhibitory effect on NLRP3, ASC and AIM2 (Figure 3A,B). We should note that KT-5823 could only slightly revert 8-Br-cGMP-induced effects (Figure 3A,B) suggesting that unregulated cGMP intracellular availability can activate signaling pathways beyond PKG-I. 

The activation of NLRP1 requires a functional cleavage of an auto-inhibitory peptide [27]. As shown in Figure 3A, resting THP-1 cells express both full length and cleaved NLRP1, as indicated by the presence of two bands in western blotting (Figure 3A). The treatment with LPS + ATP increases the intensity of both the bands whereas, in the presence of NPs and 8-Br-cGMP, the band corresponding to the full-length form completely disappears and the one corresponding to the cleaved form is strongly reduced by BNP and 8-Br-cGMP. Thus, it can be speculated that ANP and BNP do not act through the same intracellular signaling pathways. In fact, although both the NPs interfere with NLRP1 synthesis, only BNP significantly reduced NLRP1 activation. 

Inflammasome activation culminates in Caspase-1 activation/cleavage. In the same experimental conditions, we analyzed Caspase-1 activation, and found that ANP, BNP and 8-Br-cGMP inhibit Caspase-1 activation as indicated by p20 Caspase-1 fragment downregulation (Figure 3C,D). Pre-treatment with KT-5823 completely reverted the effects of NPs and 8-Br-cGMP on Caspase-1 cleavage (Figure 3C,D). Being caspase-1 the key enzyme responsible for the cleavage/maturation of IL-1β, these data further support the conclusion that NPs and 8-Br-cGMP interfere with LPS + ATP-induced IL-1β maturation and that KT-5823 abrogates NPs and 8-Br-cGMP effects on the cleavage of IL-1β (Figure 2B,E,F).

Inflammasome activation relies on a physical interaction among its components, mediated by the adaptor protein ASC which bridges inflammasome receptor and Caspase-1 [18]. Upon joining the platform, Caspase-1 get activated by a proximity-induced autoprocessing mechanism that results in the formation of the catalytically active Caspase-1 deputed to IL-1β maturation [18].

**Figure 4:**
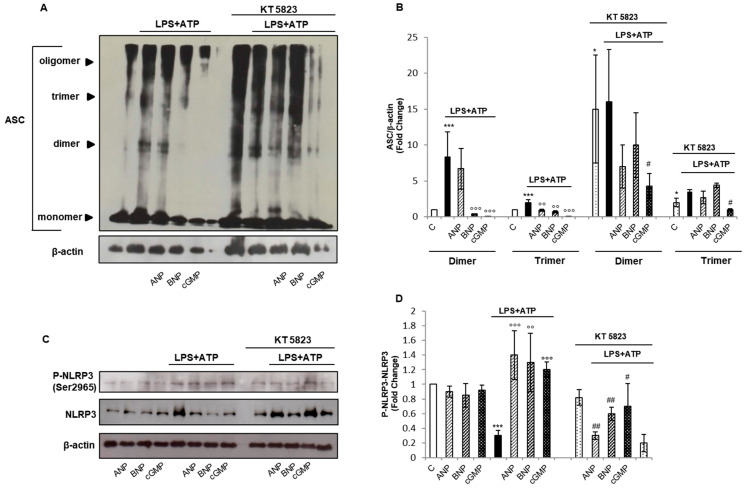
cGMP/PKG-I axis induces NLRP3 phosphorylation. THP-1 cells were pre-treated for 10 min with ANP (10^−8^ M) or BNP (10^−8^ M) or cGMP (100 µM 8-Br-cGMP) in the absence or presence of LPS (10 µg/mL for 20 min) + ATP (5 mM for 40 min). When KT-5823 (1 μM) was used, it was added 1 h before ANP, BNP, or 8-Br-cGMP treatment. (**A**) ASC oligomerization assessed by crosslinking assay followed by Western blot analysis. (**C**) Cell lysates were immunoblotted for p-NLRP3 (Ser295) or total NLRP3. The blots were stripped and re-probed with mouse anti-β-actin, to confirm equal loading. Representative western blots images are shown. (**B**,**D**) Histograms represent densitometric quantification. Bars represent the ratio between active p-NLRP3 and NLRP3 or dimeric and trimeric ASC and β-actin band intensity. Untreated cells were used as control and assumed as 1. The histogram indicates mean ± SD of at least *n* = three independent experiments, each one tested in triplicate. * *p* < 0.05, *** *p* < 0.001 versus untreated cells; °° *p* < 0.01 and °°° *p* < 0.001 versus LPS + ATP treated cells; # *p* < 0.05 and ## *p* < 0.01 versus KT-5823 + LPS + ATP treated cells.

To determine whether ANP and BNP could affect the assembly of the inflammasome platform, we performed a biochemical cross-linking experiment. We observed an augmented multimerization of ASC in the presence of LPS + ATP (Figure 4A,B). BNP and cGMP completely revert LPS + ATP-induced ASC multimerization and ANP slightly affects LPS + ATP-induced effect. These data indicate that NPs and cGMP, although to a different extent, interfere with inflammasome platform assembly. We can argue that the discrepancies between ANP and BNP effects rely on their different half-lives. ANP displays a two-minute half-life in human [31] whereas BNP half-life is of approximatively 20 min [32]. However, the known presence of neprilysin, a metalloproteinase catalyzing the degradation of NPs, on the surface of the immune system cells could affect NPs half-life [33]. Nevertheless, our hypothesis is sustained by the finding that the hydrolysis resistant cGMP analogue, 8-Br-cGMP, behaves similarly to the “long-living” BNP. 

It is known that inflammasome assembly can be abrogated by phosphorylation of NLRP3 that interrupts the interaction between NLPR3 and ASC [20].

In order to assess whether PKG-I activation could impede inflammasome assembly, we performed the crosslinking assay in the presence of KT-5823. We observed that PKG inhibition reverted ANP and BNP effects and slightly affect 8-Br-cGMP-mediated changes (Figure 4A,B). We should note that the exposure to the PKG inhibitor increases ASC multimerization, thus clearly indicating a key role of PKG-I-mediated phosphorylation as regulatory mechanism of inflammasome activation. 

We then performed a group-based phosphorylation site prediction using GSP5.0 software [34]. We found that NLRP3 protein can be phosphorylated by PKG I and II in at least six positions (Table 1). These data indicate that the cGMP/PKG-I axis might be involved in the post-translational control of inflammasome activation. 

S295, which lays in the NOD domain of NLRP3, is involved in the ATP hydrolysis that regulates NLRP3 self-oligomerization and assembly of the NLRP3-ASC complexes [35]. It has been demonstrated that S295 phosphorylation by PKA leads to a rapid NLRP3 inhibition [35]. However, Ser295 residue is also the substrate of PKD that, on the contrary, triggers inflammasome assembly [36]. This apparent discrepancy has been explained hypothesizing that a sequential phosphorylation-dephosphorylation process of S295 is required for NLRP3 inflammasome platform full activation [37]. Thus, we analyzed the involvement of PKG in the regulation of inflammasome platform aggregation via Ser295 phosphorylation (Figure 4C,D). We found that LPS, besides increasing NLRP3 expression, strongly suppressed NLRP3 phosphorylation at S295. On the other hand, pre-treatment with ANP, BNP and 8-Br-cGMP strongly enhanced NLRP3 phosphorylation. The presence of KT-5823 reduced NPs and 8-Br-cGMP mediated effects indicating that PKG-I is involved in inflammasome assembly through the phosphorylation of NLRP3 at S295. Several phosphorylation sites have been shown to regulate NLRP3 inflammasome assembly, either positively or negatively [37], but none of the potential phosphorylation sites predicted by our in-silico analysis have been described so far. 

All together, these data indicate that BNP and to a lesser extent ANP interfere with Caspase-1 activation through the cGMP/PKG-I axis which impedes the inflammasome platform assembly via NLRP3 phosphorylation at the Ser 295. The observation that ANP does not fully abrogate ASC oligomerization suggest that it might interfere with other routes of inflammasome activation to cause the inhibition of IL-1β secretion.

### 2.4. cGMP but Not PKG-I Is Involved in Non-Canonical and Alternative Inflammasome Activation

The inability of ANP to completely revert LPS+ATP-induced ASC oligomerization prompted us to analyze other routes of inflammasome activation. In fact, besides the canonical way, non-canonical and alternative routes of inflammasome activation have been reported [18,19,20].

**Figure 5:**
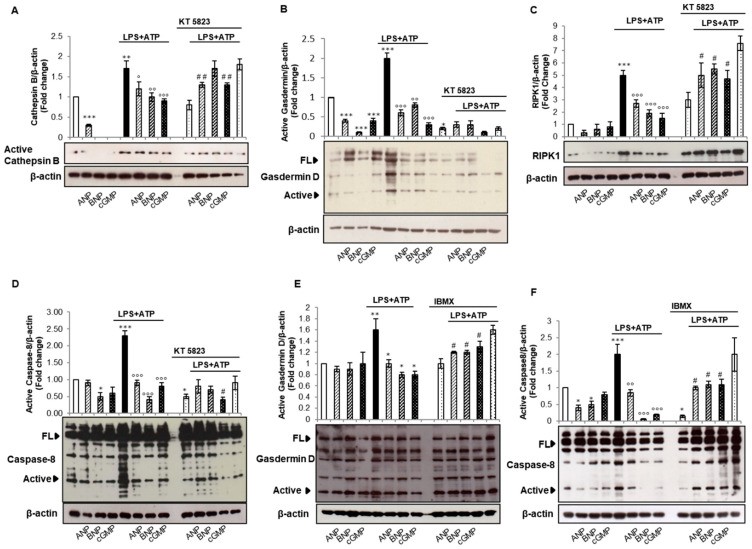
cGMP but not PKG-I is involved in non-canonical and alternative inflammasome activation. THP-1 cells were pre-treated for 10 min with ANP (10^−8^ M) or BNP (10^−8^ M) or cGMP (100 µM 8-Br-cGMP) in the absence or presence of LPS (10 µg/mL for 20 min) + ATP (5 mM for 40 min). When KT-5823 (1 μM) or 3-isobutyl-1-methylxanthine (IBMX) (10 μM) were used, they were added 1 h before ANP, BNP, or 8-Br-cGMP treatment. Cell lysates were immunoblotted for active Cathepsin B (**A**), Gasdermin D (**B**,**E**), RIPK1 (**C**), or Caspase-8 (**D**,**F**). Protein loading was assessed by re-probing the blots with an anti-β-actin antibody. One representative image is shown. Representative western blots images are shown. Histograms of densitometric quantification. Bars represent the ratio between respective protein and β-actin band intensity. Untreated cells were used as control and assumed as 1. All histograms indicate mean ± SD of at least *n* = three independent experiments, each one tested in triplicate. * *p* < 0.05, ** *p* < 0.01, *** *p* < 0.001 versus untreated cells; ° *p* < 0.05, °° *p* < 0.01 and °°° *p* < 0.001 versus LPS + ATP treated cells; # *p* < 0.05 versus KT-5823 + LPS + ATP treated cells.

As aforementioned, the canonical inflammasome activation requires two signals: the first is represented by TLR-ligand engagement; the second, provided by stress signals, results in Caspase-1 activation. Lysosomal disruption appears to be a critical event in the second step and an increase in Cathepsin B cleaved/active form has been shown to contribute to Caspase-1 activation [38]. In order to determine the involvement of ANP, BNP, and the related cGMP/PKG-I axis in canonical inflammasome activation, we analyzed Cathepsin B cleavage in our experimental conditions (Figure 5A). We found that the mature/active form of Cathepsin B is increased by LPS + ATP and that ANP, BNP, and 8-Br-cGMP diminish LPS+ATP-induced effects. Pre-incubation of THP-1 cells with KT-5823 abolished BNP-induced effects, and slightly affects ANP and 8-Br-cGMP-induced effects (Figure 5A). These results corroborate the direct involvement of cGMP/PKG-I axis induced by BNP in the canonical mechanism of inflammasome activation mediated by Cathepsin B activation. On the other hand, the effects of ANP on Cathepsin B seems to be independent of PKG-I. 

The non-canonical inflammasome signaling relies on the activation of Caspases 4 and 5 in human and Caspase 11 in rodents by a direct binding to cytosolic LPS. Differently from Caspase 1, Caspase 4/5 do not process interleukins but may induce pyroptosis. Caspase 4/5 share with Caspase 1 the pyroptotic substrate Gasdermin D [39,40], a member of the large Gasdermin family. LPS-activated Caspases 4/5 cleave Gasdermin D within the linker between N-terminal and C-terminal domains. The free N-terminal domain translocates to the plasma membrane forming pores that may cause cell swelling and osmotic lysis [39]. The Gasdermin D pore has an inner diameter of 10–14 nm and also serves as a conduit for the release of IL-1β. The N-terminal fragment of Gasdermin D also induces NLRP3-dependent activation of caspase-1 in monocytes [39,41]. To understand whether ANP- and BNP- induced cGMP/PKG-I axis affects Gasdermin D cleavage, we analyzed Gasdermin D expression and activation in our experimental conditions (Figure 5B). As expected, LPS + ATP increase the expression of both full-length and cleaved Gasdermin D. Pre-treatment with ANP, BNP, and 8-Br-cGMP strongly reduced Gasdermin D expression and activation (Figure 5B), indicating that the increase in cGMP levels elicited by NPs is involved in the inhibition of Gasdermin D. We found that also the pre-treatment with KT-5823 strongly reduced both the full-length and cleaved Gasdermin D levels in all the tested experimental conditions (Figure 5B), pointing to a direct positive contribution of PKG-I to Gasdermin D expression. 

The alternative inflammasome activation mechanism culminates, upon TLR4 engagement and Toll/IL-1 receptor domain-containing adaptor inducing IFN-β (TRIF) and receptor interacting protein kinase 1 (RIPK1) endorsement, in the cleavage of Caspase-8 [20]. To explore whether ANP, BNP, and related cGMP/PKG-I axis can disturb alternative inflammasome activation, we evaluated the expression of RIPK1 and cleaved/active Caspase-8 in our experimental conditions. We found that the exposure to LPS + ATP strongly increases the expression of RIPK1 (Figure 5C) and the cleavage of Caspase-8 (Figure 5D) and that ANP, BNP, and 8-Br-cGMP counteract LPS + ATP-induced effects (Figure 5C,D). 

It has been proposed that Caspase-8 regulates activation of NF-κB to modulate inflammation, although this function does not require Caspase-8-activating cleavage [42]. It has also been demonstrated that Caspase-8 can directly cleave pro-IL-1β or regulate the NLRP3 inflammasome [43].

Macrophages and dendritic cells from *Ripk3^−/−^Casp8^−/−^* mice demonstrate drastically reduced activation of both the canonical and noncanonical NLRP3 inflammasomes and Caspase-8 plays a role in both priming and activation of the NLRP3 inflammasome complex [44]. In particular, Caspase-8 has been detected in NLRP3 inflammasome complex, where it is involved in cleavage and processing of pro-Caspase-1 [44]. On the whole, these results suggest that NPs, by interfering with the alternative route of inflammasome activation and inhibiting Caspase-8 cleavage, regulate the entire pathway involved in IL-1β maturation and secretion. 

Pretreatment with KT-5823 affects ANP- and BNP- and 8-Br-cGMP-mediated RIPK1 expression (Figure 5C). In fact, the presence of NPs and cGMP more than halved LPS-induced RIPK1 expression, whereas in the presence of KT-5823 only a reduction of approx. 25% is observed. The presence of KT-5823 diminished LPS + ATP-induced cleavage of Caspase-8 (Figure 5D), pointing to PKG-I involvement in LPS + ATP-mediated effects downstream of RIPK1. 

These results are in agreement with the finding that nitric oxide induces a cGMP/PKG-dependent activation of Caspase-8 in *Aeromonas hydrophila*-infected head kidney macrophages of the fish *Clarias batrachus* [45]. 

To determine whether other cGMP intracellular targets could be involved in ANP- and BNP- mediated effects on non-canonical and alternative inflammasome activation routes, we pretreated THP-1 cells with IBMX (10μM) a non-specific phosphodiesterase (PDE) inhibitor (Figure 5E,F). We found that in the presence of IBMX the effects of NPs and 8-Br-cGMP on Gasdermin D and Caspase-8 cleavage was dramatically reduced (Figure 5E,F), pointing to the involvement of PDEs on NPs- induced effects on the Gasdermin D and Caspase-8 cleavage. Since PDEs mediate the crosstalk between cGMP and cAMP pathways [46] our results correlate with the finding that the activation of PKA mediated by an intracellular rise of cAMP can control cytosolic LPS-induced activation of the Caspase-11 and the consequent pyroptosis in mouse macrophages [47]. Moreover, our data suggest that NPs, through a crosstalk with the cAMP pathway, can regulate the non-canonical and alternative routes of inflammasome activation. 

Overall, these results reveal that both NPs lead to the inhibition of IL-1β secretion interfering with all the routes of inflammasome activation. However, although the increase in cGMP intracellular levels evoked by NPR-1 activation is a central mediator of NPs effects, the involvement of PKG-I seems to be route-dependent. In fact, NPs requires the cGMP/PKG-I axis to modulate the canonical route via PKG-I-dependent NLRP3 phosphorylation at Ser295, whereas other cGMP targets, such as PDEs, seem to be involved in the inhibition of the non-canonical and alternative routes of inflammasome activation. 

## 3. Materials and Methods

### 3.1. Reagents

All of the chemicals used in the present study were analytical grade reagents from various sources. Human ANP was obtained from Merck KGaA (Darmstadt, Germany) and dissolved in 5% acetic acid. Human BNP was obtained from Phoenix Europe GmbH and dissolved in H_2_O. LPS (from Escherichia coli 0111: B4), 8-Bromoguanosine 3′,5′-cyclic monophosphate sodium salt (8-Br-cGMP), ATP (dissolved in H_2_O), and the PKG inhibitor KT-5823 and the PDEs inhibitor IBMX (3-isobutyl-1-methylxanthine) (dissolved in 100 % DMSO), were purchased from Merck KGaA (Darmstadt, Germany). BS(PEG)5 (#21581) was from Thermo Fisher Scientific, (USA) and dissolved in DMSO. All the primary antibodies, unless otherwise stated were from Cell Signaling Technology (Leyden, The Netherlands). 

### 3.2. Cell Culture and Drug Treatments

**Scheme 1:**
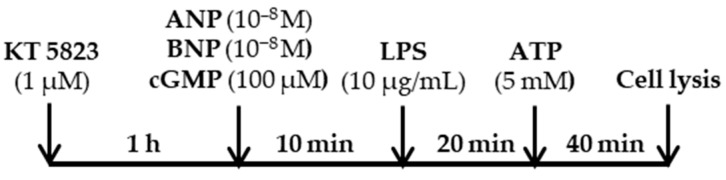
Scheme of the experimental protocol.

Human THP-1 monocytes were purchased from American Type Culture Collection (ATCC, USA) and routinely maintained at 37°C in 5% CO_2_ in RPMI 1640 supplemented with 10% heat inactivated (1 h at 56 °C) FBS, 1× l-glutamine, 1 mM sodium pyruvate, 1× non-essential amino acids, 100 units/mL of penicillin and 0.1 mg/mL of streptomycin (Invitrogen, Italy). THP-1 cells (2.5 × 10^6^ cells/well) were plated in 24-well culture dishes and pre-treated for 10 min with human ANP (10^−8^ M), human BNP (10^−8^ M) or 8-Br-cGMP (100 µM), exposed to LPS (10 µg/mL) for 20 min and then to ATP (5 mM) for the last 40 min (Scheme 1). In independent experiments, treatments were extended for 48 h. In independent experiments, KT-5823 (1 µM) or IBMX (10 μM) were added to cells 1 h before treatments (Scheme 1). DMSO or acetic acid produce no significant toxicity, therefore all the relative treatments were compared to these latter controls. 

At the end of the treatments, total cell lysates were prepared using RIPA buffer with protease and phosphatase inhibitors.

### 3.3. Measurements of Secreted IL-1β

Measurements of secreted IL-1β were performed in 100 µL supernatant after THP-1 exposure to the indicated compounds for 48 h at 37 °C. After treatments, supernatants were collected and human IL-1β levels were determined by the specific ELISA kit, according to the manufacturer’s guidelines (ThermoFisher, Waltham, MS, USA).

### 3.4. cGMP Measurements

cGMP intracellular levels were determined by the specific EIA kit, according to the manufacturer’s guidelines (GE Healthcare, Chicago, IL, USA). THP-1 cells (1 × 10^6^ cells/well) were plated in 48-well culture dishes, incubated for 30 min with human ANP or BNP (10^−8^ M) and lysed in 200 µL of the provided lysis buffer. Measurements were performed in 50 µL of the lysate.

### 3.5. Western Blot Analysis

Total proteins (15 µg) were separated by 12% sodium dodecyl sulfate-polyacrylamide gel electrophoresis (SDS-PAGE) and transferred to nitrocellulose membrane. Non-specific binding sites were blocked in Roti-Block (Roth GmbH, Nürnberg, Germany) for 1 h at room temperature. The membranes were blotted overnight at 4 °C with the following anti-human Abs diluted 1:1000 in Roti-Block: anti-NLRP3 (D4D8T) (#15101T), anti-AIM2 (D5X7K) (#12948T), anti-ASC/TMS1 (E13EI) (#13833T), anti-Phospho-NF-κB p65 (Ser536) (93H1) (#3033), anti-IL1-β (D3U3E) (#12703T), anti-Cleaved-IL1-β (Asp116) (D3A3Z) (#83186T), anti-Gasdermin D (E8G3F) (#97558) rabbit monoclonal Abs (mAb); anti-Caspase-1 (#2225), anti NLRP1 (#4990T), anti-Phospho-p44/42 MAPK (ERK1/2) (Thr202/Tyr204) (#9101), anti-RIPK1 (#4926); anti-total ERK1/2 (#9102), anti-total NF-κB p65 (#3034), rabbit polyclonal Abs (pAb); anti-Cathepsin B (#MA5-32651), anti-NPR-1 (#PA5-29049), and anti-pNLRP3 (Ser 295) (# PA5-105071), rabbit pAb (Thermo Fisher Scientific, Waltham, MS, USA), anti-Caspase 8/p43/p18 (#13423-1-AP) rabbit pAb (Proteintech, Manchester, UK). After washing with TBST, blots were incubated for 1 h at room temperature with the appropriated horse radish-peroxidase (HRP)-conjugated secondary Abs (1:2000 dilution) and revealed using the enhanced chemiluminescence (ECL) system (Merck KGaA, Darmstadt, Germany). Membranes were stripped and re-probed with anti-β-actin mAb (I-19) antibody (1:400) (Santa Cruz Biotechnology, Dallas, TX, USA) as loading control. Densitometric analyses were performed with ImageJ software.

### 3.6. Chemical Crosslinking of ASC Oligomers

Crosslinking of ASC oligomers was performed according to the manufacturer’s guidelines (Thermo Fisher Scientific, Waltham, MS, USA). Briefly, cells were resuspended in PBS (pH 7.2), subjected to five freeze and thaw cycles and centrifuged for 10 min at 10,000× *g* at 4 °C. Pellets were gently resuspended in 50 µL of PBS (pH 7.2). Subsequently, 1 µL of 250 mM BS(PEG)5 was added and incubated for 30 min at room temperature. At the end, 5 µL TRIS 500 mM (pH 7.5) was added, incubated for 15 min at room temperature and centrifuged for 10min at 10,000× *g* at 4 °C. Pellets were resuspended in 20 µL of loading buffer and resolved on a 12% SDS-PAGE and visualized by immunoblotting with the anti-ASC antibody.

### 3.7. Phosphorylation Site Prediction

Phosphorylation site prediction was performed using GPS5.0 (Group-based Prediction System, version 5.0) software (http://gps.biocuckoo.cn/index.php). The sequence of human NLRP3 protein (NP_001073289.2) in FATSA format was used as input and the search was performed with high threshold.

### 3.8. Statistical analysis

Results were expressed as means ± SD of three independent experiments performed in triplicate. The statistical significance of differences between treated and untreated cells was assessed by Student’s *t*-test. Differences between groups were considered significant when *p* < 0.05.

## 4. Conclusions

The involvement of the cardiac hormones ANP and BNP on immune system regulation in heart failure has been wildly investigated [48]. NPs, largely produced by cardiac myocytes, increase during heart failure and the inhibition of NPs degradation, also explored by the PARADIGM-HF trial, has been suggested to increase survival in heart failure patients [49]. Moreover, vericiguat, a novel oral soluble guanylate cyclase stimulator, is on a phase III clinical trial (VICTORIA; NCT02861543- last update 14 July 2020), which aims to decrease cardiovascular death and heart failure hospitalization in patients with heart failure with reduced ejection fraction [50]. 

**Figure 6:**
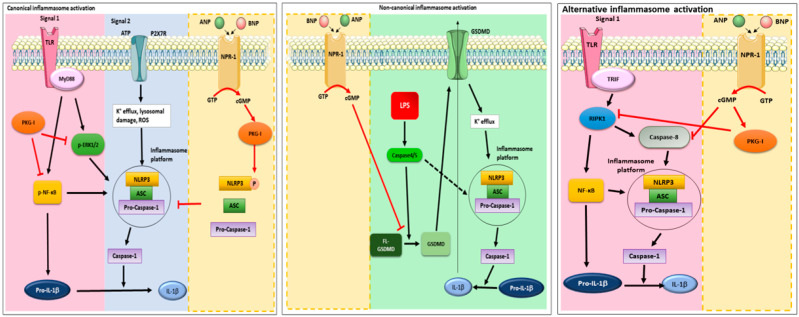
NPs interfere with canonical, non-canonical and alternative routes of inflammasome activation. Canonical inflammasome activation requires two signals: the first, represented by TLR-ligand engagement, involves NF-κB and ERK1/2 activation and culminates in the increased expression of pro-IL-1β and PRRs, such as NLRP3; the second, provided by stress signals, results in Caspase-1 activation and pro-IL-1β cleavage. NPs, by binding to NPR-1 induce an intracellular increase in cGMP which activates PKG-I. This latter is responsible for NF-κB and ERK1/2 inhibition and possibly for the phosphorylation of NLRP3, thus impeding the assembly of the inflammasome platform. Non-canonical inflammasome signaling depends on the activation of Caspase-4 and -5 in human by a direct binding to cytosolic LPS. Caspase-4/5 process Gasdermin D which translocates to the plasma membrane forming pores that may cause K+ efflux responsible for inflammasome platform assembly. Moreover, Gasdermin D may serve as a conduit for the release of IL-1β. NPs-induced increase in intracellular cGMP levels inhibits Gasdermin D cleavage. Alternative inflammasome activation pathway culminates, upon TLR4 engagement, TRIF and RIPK1 endorsement, in the cleavage of Caspase-8 which can directly cleave pro-IL-1β or regulate the NLRP3 inflammasome. PKG-I, activated by cGMP, reduced RIPK1 expression levels. Moreover, the intracellular cGMP rise evoked by NPs leads to inhibition of Caspase-8 cleavage.

In this study, we provided evidence on the molecular mechanism underlying ANP and BNP anti-inflammatory and immunomodulatory action in human THP-1 cells (Figure 6). We have demonstrated that the reduction in IL-1β secretion, elicited by ANP and BNP, depends on their binding to NPR-1 leading to an increase in cGMP intracellular levels. The rise in intracellular cGMP concentration is responsible for the control of canonical, non-canonical, and alternative mechanism of inflammasome activation. 

The increase in intracellular cGMP levels may culminate in PKG-I activation [2,51]. We found that the cGMP/PKG-I axis plays a central role in both the priming and activation steps of canonical inflammasome activation. Moreover, we found that NPs-mediated cGMP/PKG-I axis activation dampen inflammasome platform assembly through a direct phosphorylation of NLRP3 at Ser295. To the best of our knowledge, this is the first report showing that post-translational modification of the inflammasome components might be mediated by PKG-I and pave the way for the conception of new pharmacological interventions aimed at targeting inflammasome activation and IL-1β secretion. 

Furthermore, we reported for the first time that both ANP and BNP, via cGMP-dependent activation of PDEs, interfere with non-canonical and alternative routes of inflammasome activation. Although the canonical, non-canonical, and alternative inflammasomes are activated in different ways, they functionally cooperate to induce the inflammatory responses. In fact, a functional crosstalk of caspase-11 and NLRP3 inflammasomes has recently been reported [52] and an amplification of the inflammatory response by cooperation between Caspase-8 and Caspase-11 has been suggested [53]. Interestingly, non-canonical inflammasome activation pathway has been explored in search for new potential therapeutic targets for cardiovascular diseases [54]. The importance of IL-1β dysregulation in cardiac pathologies is further supported by the CANTOS trial (CANTOS ClinicalTrials.gov number, NCT0132784). It demonstrated that the anti-inflammatory therapy targeting IL-1β with canakinumab (an IL-1β blocking antibody) led to a significantly lower rate of recurrent cardiovascular events [55]. It is also to highlight that the NLRP3 inflammasome in macrophages can be activated by a multitude of viruses and viral proteins, such as the severe acute respiratory syndrome coronavirus (SARS-CoV) viroporin [56] and that the Italian agency of pharmaceuticals (AIFA) recently approved the use of canakinumab for the treatment of patients affected by Coronavirus disease-19 (COVID-19) (aifa.gov.it). Thus, NPs might be proposed not only as a treatment for cardiac pathologies, but also to prevent and treat the plethora of diseases caused by a dysregulated inflammasome activation.

## Figures and Tables

**Table 1:** Potential sites of PKG I and II -mediated phosphorylation in NLRP3 protein.

Position	Code	Kinase	Peptide	Score	Cutoff
169	T	AGC/PKG/PRKG1	VSLNKRYTRLRLIKE	10,511	10,046
295	S	AGC/PKG/PRKG1	HKIVRKPSRILFLMD	11,906	10,046
955	S	AGC/PKG/PRKG1	SQSLRKLSLGNNDLG	12,266	10,046
102	S	AGC/PKG/PRKG2	GSDNARVSNPTVICQ	64.2	55,934
295	S	AGC/PKG/PRKG2	HKIVRKPSRILFLMD	59,621	55,934
436	S	AGC/PKG/PRKG2	GKSLAQTSKTTTAVY	60,347	55,934
728	S	AGC/PKG/PRKG2	VNSHLTSSFCRGLFS	56,211	55,934
955	S	AGC/PKG/PRKG2	SQSLRKLSLGNNDLG	57,236	55,934

**Position**: The position of the site which is predicted to be phosphorylated. **Code**: The residue which is predicted to be phosphorylated. **Kinase**: The regulatory kinase which is predicted to phosphorylate the site. **Peptide**: The predicted phosphopeptide with 7 amino acids upstream and 7 amino acids downstream around the modified residue. **Score**: The value calculated by GPS algorithm to evaluate the potential of phosphorylation. The higher the value, the more potential the residue is phosphorylated. **Cutoff**: The cutoff value under the threshold. Different threshold means different precision, sensitivity and specificity. In red the predicted phosphorylation site.

## Data Availability

Data are contained within the article or supplementary material.
